# Consumption of Coffee and Tea Is Associated with Macular Retinal Nerve Fiber Layer Thickness: Results from the UK Biobank

**DOI:** 10.3390/nu15051196

**Published:** 2023-02-27

**Authors:** Yixiong Yuan, Gabriella Bulloch, Shiran Zhang, Yanping Chen, Shaopeng Yang, Wei Wang, Zhuoting Zhu, Mingguang He

**Affiliations:** 1 State Key Laboratory of Ophthalmology, Zhongshan Ophthalmic Center, Sun Yat-sen University, Guangdong Provincial Key Laboratory of Ophthalmology and Visual Science, Guangdong Provincial Clinical Research Center for Ocular Diseases, Guangzhou 510060, China; 2 Centre for Eye Research Australia, Royal Victorian Eye and Ear Hospital, Melbourne, VIC 3002, Australia; 3 Ophthalmology, Department of Surgery, University of Melbourne, Melbourne, VIC 3010, Australia; 4 Department of Ophthalmology, Guangdong Eye Institute, Guangdong Provincial People’s Hospital, Guangdong Academy of Medical Sciences, Guangzhou 510080, China

**Keywords:** coffee, tea, neurodegeneration, optical coherence tomography

## Abstract

Coffee and tea drinking are thought to be protective for the development and progression of neurodegenerative disorders. This study aims to investigate associations between coffee and tea consumption with macular retinal nerve fiber layer (mRNFL) thickness, a marker of neurodegeneration. After quality control and eligibility screening, 35,557 out of 67,321 United Kingdom (UK) Biobank participants from six assessment centers were included in this cross-sectional study. In the touchscreen questionnaire, participants were asked how many cups of coffee and tea were consumed daily on average over the last year. Self-reported coffee and tea consumption were divided into four categories including 0 cup/day, 0.5–1 cups/day, 2–3 cups/day, and ≥4 cups/day, respectively. The mRNFL thickness was measured by the optical coherence tomography (Topcon 3D OCT-1000 Mark II) and automatically analyzed by segmentation algorithms. After adjusting for covariates, coffee consumption was significantly associated with an increased mRNFL thickness (β = 0.13, 95% CI = 0.01~0.25), which was more prominent in those who drank 2~3 cups coffee per day (β = 0.16, 95% CI = 0.03~0.30). The mRNFL thickness was also significantly increased in tea drinkers (β = 0.13, 95% CI = 0.01~0.26), especially for those who drank more than 4 cups of tea per day (β = 0.15, 95% CI = 0.01~0.29). The positive associations with mRNFL thickness, indicating that both coffee and tea consumptions had likely neuroprotective potentials. Causal links and underlying mechanisms for these associations should be explored further.

## 1. Introduction

Coffee and tea have been enjoyed for centuries around the world [1], and it is estimated that more than ten and six billion tons of coffee [2] and tea [3] are consumed worldwide in 2021. Considering their volume of intake, if coffee or tea had any positive or negative medical benefit, they would impact public health enormously.

Both coffee and tea are known to contain caffeine which is best known for its stimulating effects on cognition, attention, and wakefulness [4]. The discovery of other antioxidants such as flavonoids and polyphenols in coffee and tea [5] have led to the hypothesis that their intake may have neuroprotective benefits. Epidemiological studies have discovered coffee and tea drinkers have reduced odds of Parkinson’s disease [6] and dementia [7] but unfortunately medical evidence to support these benefits are limited [8]. To complicate things further, magnetic resonance imaging (MRI) studies show inconsistent results when comparing brain volume between consumers and non-consumers [9,10,11].

The retina is a tissue that extends from the central nervous system (CNS) and presents itself as a unique window for non-invasively detecting brain and vascular disease through optical coherence tomography (OCT) [12]. OCT studies indicate that retinal nerve fiber layer (RNFL) thinning is significantly associated with cognition [13], and states of neurodegeneration such as glaucoma [14], Parkinson’s disease [15], Alzheimer’s disease [16], and mild cognitive impairment [17]. With OCT now being performed routinely in hospital and community settings, and its ability to image the RNFL at a micron level, OCT’s potential dual purpose as a risk-stratification tool for neurodegenerative states should be considered [18].

Given that the RNFL reflects neurodegenerative changes, we intend to investigate associations between self-reported coffee and tea consumptions with OCT-measured macular retinal nerve fiber layer (mRNFL) thickness in a subgroup of United Kingdom (UK) Biobank participants. The combination of self-reported information and objective retinal measurements might bring additional evidence to support the neuroprotective potentials of these two beverages and provide novel insight into the prevention and treatment of neurodegenerative disorders. 

## 2. Materials and Methods

### 2.1. Study Population

The UK Biobank is a population-based cohort study, with more than half a million participants recruited from England, Scotland, and Wales. All participants were aged 40–69 years old at the time of recruitment and lived within twenty-five miles of assessment centers. The baseline visit (2006–2010) consisted of touchscreen questionnaires, verbal interviews, physical measurements, blood, and urine assays. From June 2009 to July 2010, a subgroup of participants from six designated assessment centers (Sheffield, Liverpool, Hounslow, Croydon, Birmingham, and Swansea) were invited to receive additional eye examinations including intraocular pressure, autorefraction, visual acuity, and macular OCT at baseline. The UK Biobank was conducted with ethics approval from the National Information Governance Board for Health and Social Care and North West Multicenter Research Ethics Committee (11/NW/0382), and was carried out in accordance with the Declaration of Helsinki. Informed consents and authorizations to access anonymous health records were obtained from all participants. Deidentified data were stored in the UK Biobank database, with personal identifiers kept separately under strict control with restricted access.

Participants who completed baseline OCT measurements were included in this study. According to established standards for quality control, eyes with low signal strength (Q < 45), weak centration or segmentation indicators (poorest 20%) were excluded from analyses. To avoid interference from other ocular parameters, eyes with high refractive error (spherical equivalent [SE] >6 or <−6 diopters [D]), visual impairment (>0.1 logarithm of the minimum angle of resolution [logMAR]), or abnormal intraocular pressure (IOP) (≥22 or ≤5 mmHg) were also excluded. Considering the probable RNFL destructions secondary to neurodegenerative diseases, patients with glaucoma, other retinal disorders, multiple sclerosis, dementia, and Parkinson’s diseases were identified from participants’ medical history consisting of questionnaires, interviews, and inpatient diagnoses before baseline (Detailed in Table S1) and excluded. In addition, participants refusing to answer questions about coffee or tea consumption were further excluded. As for those with both eyes being deemed high quality, one eye was selected for analysis. 

### 2.2. Coffee and Tea Consumption

In the baseline questionnaire, participants were asked how many cups of coffee and tea were consumed daily on average over the last year. Participants’ answers were limited in the range of 0 to 99, and those who consumed more than ten cups of coffee or tea per day were required to confirm the accuracy of their answers. In the current study, daily amounts of coffee and tea consumption were further divided into four categories; 0 cup/day, 0.5–1 cups/day, 2–3 cups/day, and ≥4 cups/day. Additionally, coffee drinkers were asked which type of coffee was usually consumed or consumed the most. Participants accustomed to drinking instant coffee were categorized separately. Type of tea was not implicated in the touchscreen questionnaire.

### 2.3. Eye Examinations and OCT Measurements

**Figure 1:**
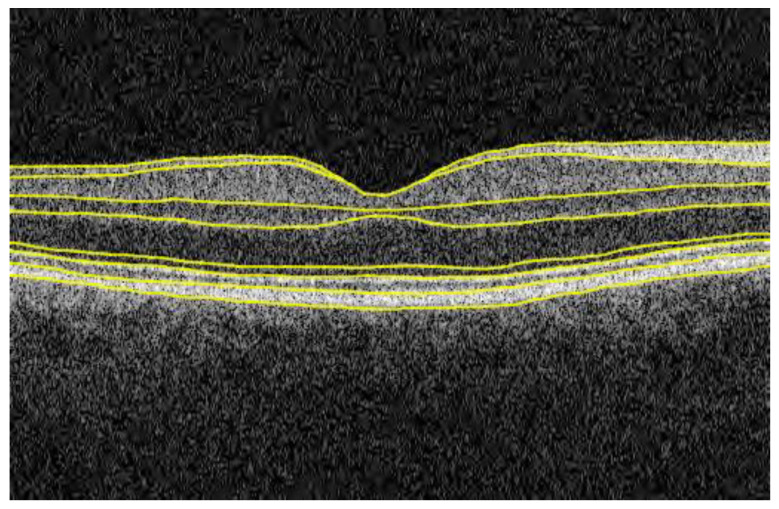
Schematic diagram of macular retinal nerve fiber layer in spectral-domain optical coherence tomography images. he top two lines refer to the inner limiting membrane and inner surface of ganglion cell layer. Areas between these two lines refer to the macular retinal nerve fiber layer.

All participants in this study underwent visual acuity, autorefraction, IOP, and macular OCT measurements. Examinations were performed on both eyes, beginning with the right. Visual acuity was tested with 4-metre traditional LogMAR charts with refractive correction (spectacles or contact lens), if any. The refractive error was measured by autorefraction (Tomey RC5000; Nagoya, Japan). SE values were calculated based on autorefraction results (sphere degree + 0.5 × cylinder degree). IOP values were measured by the Ocular Response Analyzer (ORA, Reichert, Corp., Buffalo, NY, USA) which consisted of two consecutive measurements in one single test. The corneal-compensated IOP, a linear combination of the two measurements, was recommended by the previous literature and used in the current study [19]. Commercial spectral-domain OCT (Topcon 3D OCT-1000 Mark II; Topcon, Inc., Tokyo, Japan) obtained 6 × 6 mm^2^ macular volume scans on non-dilated eyes in dark rooms, with axial resolution of 6 µm. Each volume scan consisted of 512 A-scans × 128 B-scans and required about 3.7 s (18,000 A-scans/second). Six assessment centers used the same model of OCT devices and at least three trained technicians were assigned to each center. After acquisition, OCT images were submitted to UK Biobank servers and stored in a central repository. A custom image segmentation software, Topcon Advanced Boundary Segmentation (TABS) algorithm Version 1.6.1.1 (Topcon Advanced Biomedical Imaging Laboratory, Oakland, CA, USA), was used to perform automated location of fovea and segmentation of retinal layers. Validity (Overall border position differences: 0.82~3.45 µm) and reliability (Intraclass correlation: 0.942–0.993) of the TABS algorithm were reported in previous studies [20]. The mRNFL referred to bright zones between the inner limiting membrane and ganglion cell layer (Figure 1). In this study, the average mRNFL thickness across six subfields (superior, superior-temporal, superior-nasal, inferior, inferior-nasal, and inferior-temporal) was analyzed.

### 2.4. Covariates

To control for potentially confounding variables, demographic, socioeconomic, lifestyle, and health-related covariates were included in this study. In brief, age at baseline assessment were divided into five categories, including <50 years, 50–54 years, 55–59 years, 60–64 years, and >64 years. The UK Biobank assessment center at which participant attended were automatically acquired. Townsend deprivation index (TDI) were assigned according to participants’ postal codes, which reflected the proportions of unemployment, crowding household, non-car ownership, and homelessness in corresponding output areas. Four quantiles were categorized in the ascending order for TDI (<−3.6, −3.6~−2.1, −2.1~0.6, and >0.6). Body mass index (BMI) were constructed from height and weight, which were measured by Seca 240 cm height measure (Seca Gmbh & Co. KG., Hamburg, Germany) and Tanita BC418MA body composition analyzer (Tanita Corp., Tokyo, Japan) at baseline respectively. In the touchscreen questionnaire, UK Biobank participants were asked about their ethnic group, including White, Mixed, Asian, Black, Chinese. Due to the small number of participants, the last five alternatives were assembled into others. The average total household income before tax were directly derived from questionnaires, including <GBP 18,000, GBP 18,000 to GBP 30,999, GBP 31,000 to GBP 51,999, GBP 52,000 to GBP 100,000, and >GBP 100,000. Educational qualifications reflected the highest diploma achieved which were divided into three categories including O levels or equivalent, A levels or equivalent, and college or university degree. Time spent on moderate to vigorous activity (MVPA) was categorized into four quantiles based on adapted questions from the short International Physical Activity Questionnaire [21]. Weighted by expended energy, MVPA time were transformed into metabolic Equivalent Task (MET) minutes/week and categorized into four quantiles. Sleep duration was derived from the average hours spent on both nocturnal sleep and daytime naps for a 24 h day in the last 4 weeks, which were further divided into four categories including <7 h, 7 h, 8 h, and >8 h. For smoking status, previous smokers and current smokers were distinguished from those who never smoked tobacco. Similarly, previous and current drinkers were also separated from those who never consumed alcohol. According to the consumption of different foods over the last year, diet patterns were determined as healthy or unhealthy diet in accordance with previous studies [7]. Seven components of healthy diet were defined (fruits, vegetables, and whole grains ≥ 3 servings/day; fish ≥ 2 servings/week; unprocessed red meats and refined grains ≤ 1.5 servings/week; processed meats ≤ 1 serving/week). Participants who met the definitions of four or more components were considered to have a healthy diet. In addition, habitual intake of sweeten beverages or foods were determined based on participants’ replies and included in this study. For health-related covariates, systemic comorbidities including cardiovascular diseases, hypertension, and diabetes which were likely associated with coffee and tea consumption were also identified based on participants’ inpatient records before baseline using International Classification of Diseases-10 (ICD-10) codes. As the complement to inpatient records, systemic comorbidities were also identified if there were corresponding medical history in the touchscreen questionnaire or verbal interview. Using non-fasting venous blood samples, baseline high density liptein (HDL) cholesterol and low density liptein (LDL) cholesterol concentrations were analyzed by Beckman Coulter AU5800 (Beckman Coulter Inc., Brea, CA, USA). Participants with excessive low HDL cholesterol level (<1.04 mmol/L) and high LDL cholesterol level (>3.37 mmol/L) were categorized into abnormal groups. For covariates containing missing or unavailable values, an independent category was set and kept in the analysis. All UK Biobank fields used to retrieve baseline covariates are described in Table S1.

### 2.5. Study Limitations

The main weakness of this study was its retrospective and cross-sectional design, which limited any casual inference. Self-reported coffee and tea consumption obtained from participants’ questionnaires determined that recall bias was inevitable and it was difficult to quantify the exact intake of caffeine or other antioxidants using precise units such as milligram. This limitation also existed in other covariates derived from questionnaires and interviews. Furthermore, the outcome, mRNFL thickness, was measured by different devices and examiners in six assessment centers. Despite standardized training and supervision, the distributed measurement might still magnify random errors and weaken statistical significance.

### 2.6. Statistical Analyses

Baseline characteristics were expressed as number (percentage) for categorical covariates and mean (standard deviation, SD) for continuous covariates. Chi-square tests, Student t-tests, and analyses of variance compared categorical and continuous characteristics among participants with differing frequency of coffee and tea consumption (0, 0.5–1, 2–3 and ≥4 cups/day). After adjusting for demographics, socioeconomic, medical factors, ocular parameters, and lifestyle, multivariable linear regression models evaluated the association of coffee and tea consumption with average mRNFL thickness, respectively. Due to the addition of non-dairy creamers and hydrogenated vegetable oils in some instant coffee ingredient lists, it was a concern that instant coffee could contain more trans fatty acids (TFA), which is associated with numerous systemic diseases [22]. Therefore, regular intake of instant coffee was further included in the multivariable models as a confounding factor. Restricted cubic spline (RCS) model explored potential non-linear associations between coffee and tea consumption (cups/day) with mRNFL thickness, with three knots at the 10th, 50th, and 90th percentiles. Sensitivity analyses were performed in different age subgroups (≤60 and >60 years) and gender subgroups (female and male). All *p* values were two-sided and significance was considered when *p* < 0.05. All statistical analyses were carried out using STATA 15.1 (StataCrop, College Station, TX, USA).

## 3. Results

**Figure 2:**
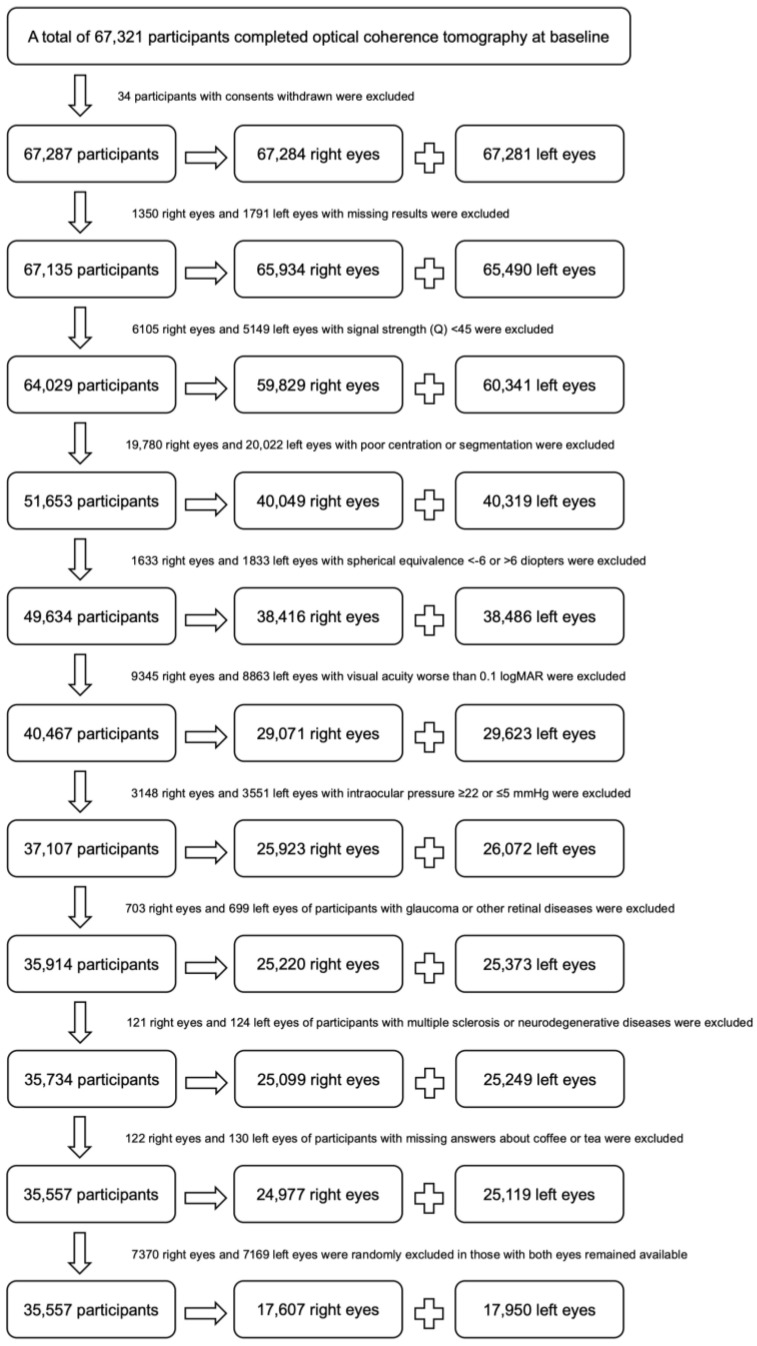
Flowchart to demonstrate the eligibility criteria.

Of the 67,321 participants who completed OCT examinations at baseline, mRNFL thicknesses were available in 67,135 participants. After quality control and exclusion of diseases which may cause mRNFL thinning, a total of 35,557 eligible participants were included in this study (Figure 2). Coffee and tea were consumed by 78% and 86% of participants respectively. Distributions of covariates across coffee and tea drinkers, and non-coffee and non-coffee drinkers are outlined in Table 1. Comparison of covariates stratified by daily cups of coffee and tea are provided in Table S2. Most covariates were significantly different between coffee and non-coffee drinkers, except for CVD and SE. As for tea drinkers, no significant differences were detectable among sex, income, ethnic background, education achievement, diabetes, CVD, hypertension, LDL, SE, and IOP with reference to non-tea drinkers. 

Multivariable linear models found that coffee consumption was not associated with mRNFL thickness after adjusting for demographic (age, sex, and assessment center) and socioeconomic covariates (TDI, household income, ethnic background, and educational qualification) in Model 1 (Table 2). Further adjustments in life-style and health-related covariates in Model 2 confirmed no significant association between coffee drinking and mRNFL thickness (Table 2). In Model 3, coffee consumption was found to be significantly associated with an increased mRNFL thickness (β = 0.13, 95% CI = 0.01~0.25; Table 3). Within coffee drinkers, the association with mRNFL thickness was only significant in those who drank 2~3 cups of coffee per day (β = 0.16, 95% CI = 0.03~0.30). These findings were supported by RCS models (Figure 2), with an inverted U-shape association found between coffee drinking and mRNFL thickness (*p* for non-linear = 0.01). Sensitivity analyses (Tables S3 and S4) indicated associations between coffee consumption and mRNFL thickness was not affected by age and gender groups (All *p* for interaction > 0.05).

Comparison of participant characteristics stratified by habitual intake of instant coffee is provided in Table S5. The mRNFL thickness was significantly thinner in instant coffee drinkers than those that did not drink instant coffee (28.36 µm vs. 28.63 µm, *p* < 0.05). On the basis of Model 2, multivariable linear models further took into account the habitual intake of instant coffee (Model 3; Table 3), which was significantly associated with a reduced mRNFL thickness (β = −0.19, 95% CI = −0.29~−0.10).

**Figure 3:**
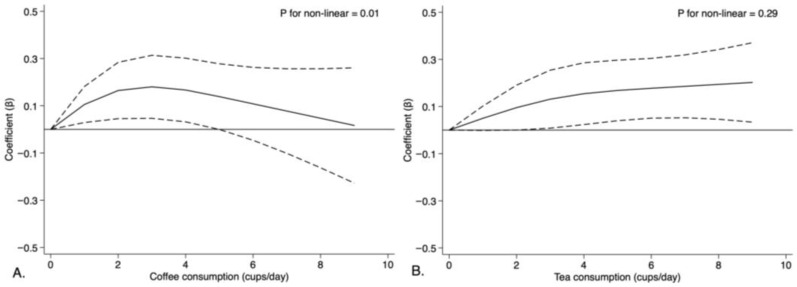
Restricted cubic spline models for the association between coffee and tea consumption with the average thickness of macular retinal nerve fiber layer. Adjusted for age at baseline, sex, assessment center, average total household income before tax, Townsend deprivation index, smoking statue, drinking statue, ethnic background, education achievement, body mass index, moderate to vigorous physical activity time, sleep duration, diabetes, cardiovascular diseases, hypertension, healthy diet, habitual intake of sweeten beverages or foods, serum high density liptein cholesterol level, serum low density liptein cholesterol level, spherical equivalent, intraocular pressure, habitual intake of instant coffee. Both coffee consumption and tea consumption were included in the restricted cubic model. The solid line represented adjusted coefficients and the dash line represented 95% confidence intervals (95% CI).

In Model 1 adjusting for demographic and socioeconomic factors, tea consumption was associated with an increased mRNFL thickness (β = 0.17, 95% confidence intervals [95% CI] = 0.04~0.29), particularly in those who consumed ≥4 cups of tea per day (β = 0.16, 95% CI = 0.01~0.30) (Table 2). Its association with mRNFL thickness remained statistically significant in Model 2 (β = 0.14, 95% CI = 0.01~0.26). Tea consumption was also significantly associated with an increased mRNFL thickness in Model 3 (β = 0.13, 95% CI = 0.01~0.26), and particularly for those who consumed ≥4 cups of tea per day (β = 0.15, 95% CI = 0.01~0.29, *p* for trend = 0.03). The RCS model (Figure 3) indicated mRNFL thickness linearly increased with tea intake (*p* for non-linear = 0.29). Sensitivity analysis (Tables S3 and S4) indicated associations between tea consumption and mRNFL thickness were not affected by age and gender groups (All *p* for interaction > 0.05).

No significant interaction was observed between coffee and tea consumption with the mRNFL thickness in Model 3 (*p* for interaction = 0.84).

## 4. Discussion

Based on OCT measurements, this study is the first to link self-reported coffee and tea consumption with retinal markers of neurodegeneration in a large real-world population. Our results demonstrate mRNFL was significantly thicker amongst coffee and tea drinkers, which was most prominent in participants drinking 2~3 cups of coffee per day and four or more cups of tea per day. In particular, an inverted U-shape association was observed between daily coffee consumption and mRNFL thickness in RCS analysis. We also found that the intake of instant coffee was associated with a reduced mRNFL thickness, which was independent from the magnitude of coffee and tea consumption. The significant findings support neuroprotective potentials of these two beverages and warrant further studies to validate their effects on neurodegenerative diseases.

Considering that mRNFL correlates with neurodegenerative states [14,15,16] and cognition [17], this study provides evidence for the assertion that coffee and tea may have neuroprotective potential. As earlier stated, 2–3 cups of coffee per day or >4 cups of tea were significantly associated with thicker mRNFL. This is in accordance with numerous previous studies, including a population-based cohort in Finland that reported a 60% risk reduction for Parkinson’s diseases in participants who drank more than five cups of coffee or three cups of tea per day [6]. The inverted U-shape association between coffee and mRNFL thickness further corroborates results from a previous meta-analysis, with the lowest risk of Parkinson’s diseases found in those who drank three cups of coffee per day [23]. In addition, large-scale studies in Japan [24] and the UK [7] observed a lower risk of dementia for moderate coffee and tea drinkers. Despite reduced risks of neurodegenerative disease, associations with brain imaging findings are still controversial. This may be attributed to the small sample sizes in these studies, resolution of the MRI, and heterogeneity among different measurement modalities [9,10,11].

Contrary to the increased mRNFL thickness in coffee drinkers, the current study found that instant coffee was negatively associated with mRNFL thickness. Overall, this study suggests that the intake of instant coffee increases risk for glaucoma, and other neurodegenerative disorders such as dementia and Parkinson’s diseases. Especially for glaucoma, coffee drinking has conflicting evidence regarding neuroprotective functions [8], which is comparison with the beneficial effect of tea leaves consistently reported in previous studies [25,26]. In Korean coffee drinkers, Bae et al. observed a 2.4 greater risk for open angle glaucoma [25] which agrees with the results of Li et al.’s UK Biobank Medelian study [27]. In contrast, Kim et al. failed to observe any significant association despite using participants from the same cohort as Li et al. [28]. Additionally, some studies indicated that caffeine intake increased the resistance of blood vessels and reduced ocular blood flow, which also could exacerbate glaucomatous optic neurodegeneration [29,30].

Trans-fatty acids (TFAs) and acrylamide (a carcinogen) have been associated with cognitive change in the past and are known to be present in some premixed coffee and instant coffee powders, but not in tea leaves. It is our opinion that their presence may account for the findings observed in instant coffee, and explain inconsistencies reported by previous studies about glaucoma [27,28]. It has been reported that TFA and acrylamide are facilitators of neurodegeneration as evidenced by human exposure cases [31,32] and animal studies with high grade exposures [33]. While this is a possible hypothesis, the few existing observational studies involving instant coffee suggest coffee is neuroprotective [7], and lab studies observe instant [34] and brewed coffee [35] reduce amyloid production. The only study suggesting adverse effects of instant coffee on cognitive impairment was in coffee drinkers who had >6 cups per day, but excessive intake of other types of coffee had similar associations [36]. Unfortunately, most studies assessing for the impact of coffee intake on degenerative diseases do not stratify for instant coffee intake, so it is difficult to contextualize these findings. Nonetheless, this large-scale study suggests instant coffee drinkers should be defined, as they are observed to impact mRNFL, and likely impact the direction of an association with neurodegenerative diseases. Importantly, these findings may have implications to public health considering coffee and tea intake occur daily and this study suggests it could have concerning implications for instant coffee drinkers. 

Several explanations for the protective potentials of coffee and tea on mRNFL thickness are worth exploring. First, the intake of caffeine regulates activation of microglia and inhibit excessive neuroinflammation which plays important roles in the development of neurodegenerative diseases [37]. Maderia et al. found caffeine-attenuated microglia-mediated inflammatory responses and reduced rat RGC loss following acute ocular hypertension insults [38]. In addition, anti-oxidants in coffee and tea are protective against reactive oxygen species and prevents ischemia-related neurodegeneration. For example, both chlorogenic acids and catechin polyphenols which are extracts from coffee and tea, alleviated oxidative-induced RGC apoptosis in rodent ischemic models [39,40]. Furthermore, catechin polyphenols extracted from green tea, especially epigallocatechin gallate, inhibited atherosclerosis [41], regulated blood lipids, and prevented insulin resistance [42]. These maintain hemostasis and lower the vulnerability of the brain to neuroinflammatory change. As the retina is an extension of the brain, these components in coffee and tea likely impact mRNFL thickness, although it is difficult to distinguish whether these theories can be related to humans as currently only laboratory studies can provide causal insight to the effects of coffee and tea on neurodegenerative changes.

To the best of our knowledge, this is the first comprehensive investigation into associations between coffee and tea consumption with mRNFL thickness, which supports the neuroprotective potentials of these two beverages from the perspective of retinal integrity. Strengths of the current study lies in its combination of real-world coffee and tea consumption with the retinal marker for neurodegenerative changes, which could be conveniently and objectively measured in large-scale population by OCT examinations. However, there are still several weaknesses in this study. First, the cross-sectional study design inhibits the formation of causal conclusions and might lead to confounding bias. Therefore, the current findings should be further validated in longitudinal studies and clinical trials. Second, it was difficult to standardize the exact amount of a cup of coffee or tea in self-reported questionnaires, which suffered from recall bias. Although coffee and tea intake were unlikely to change greatly day to day, their quantitative analyses were still limited by the lack of serum and urine caffeine concentrations or other active ingredients. Third, OCT examinations were completed by different devices and technicians in distinct assessment centers, which would magnify the measurement error and weaken possible associations with mRNFL. Although all technicians underwent structured training for OCT image acquisition and experienced ophthalmologists were responsible for quality control, this study further adjusted the assessment center as covariates to control potential bias in OCT measurements. Fourth, we could not account for the subclinical retinal and neurodegenerative diseases. Due to the lack of ophthalmic and neurological examinations, normal tension glaucoma and other insidious diseases could be misdiagnosed in self-reported questionnaires and interviews. To avoid selection bias, history of inpatient diagnosis was also considered in this study. Last but not least, it should be noted that eligible participants were selected from a subgroup of the UK Biobank population who completed additional eye examinations at baseline, which made current findings less generalizable to general populations. Furthermore, most participants were Caucasian and came from the UK. Ideally, the study should be repeated in other geographic regions and among various ethnicities to determine a universal link. 

## 5. Conclusions

In summary, this study suggests the intake of coffee and tea are associated with increased mRNFL thickness. These associations were significant in those who consumed 2–3 cups of coffee and ≥4 cups of tea daily. In contrast, mRNFL was significantly thinner in instant coffee drinkers, which highlights the need for future associative studies to adjust for coffee types. Overall, this study provides novel evidence on the neuroprotective function of coffee and tea. The roles of these two beverages for the prevention and treatment of neurodegenerative diseases should be explored.

## Figures and Tables

**Table 1:** Characteristics of coffee drinkers, tea drinkers and those who did not drink coffee or tea.

Variables	Total	Non-Coffee Drinkers	Coffee Drinkers	Non-Tea Drinkers	Tea Drinkers
	35,557 (100%)	7695 (100%)	27,862 (100%)	5082 (100%)	30,475 (100%)
Average thickness of macular retinal nerve fiber layer (µm) ^#^					
	28.52 (4.24)	28.47 (4.17)	28.53 (4.27)	28.44 (4.25)	28.53 (4.24)
Age at baseline (Years) ^§,†,‡^					
<50	9844 (27%)	2554 (33%)	7290 (26%)	1721 (34%)	8123 (27%)
50–54	5615 (15%)	1300 (17%)	4315 (15%)	767 (15%)	4848 (16%)
55–59	6212 (17%)	1351 (18%)	4861 (17%)	837 (16%)	5375 (18%)
60–64	8146 (22%)	1451 (19%)	6695 (24%)	1065 (21%)	7081 (23%)
>64	5740 (16%)	1039 (14%)	4701 (17%)	692 (14%)	5048 (17%)
Sex ^§,†^					
Female	18,952 (53%)	4405 (57%)	14,547 (52%)	2761 (54%)	16,191 (53%)
Male	16,605 (46%)	3290 (43%)	13,315 (48%)	2321 (46%)	14,284 (47%)
Assessment center ^§,†,‡^					
Sheffield	9571 (26%)	1946 (25%)	7625 (27%)	1448 (28%)	8123 (27%)
Liverpool	2628 (7%)	563 (7%)	2065 (7%)	379 (7%)	2249 (7%)
Hounslow	6867 (19%)	1506 (2%)	5361 (19%)	918 (18%)	5949 (2%)
Croydon	8935 (25%)	1901 (25%)	7034 (25%)	1231 (24%)	7704 (25%)
Birmingham	7476 (21%)	1764 (23%)	5712 (21%)	1093 (22%)	6383 (21%)
Swansea	80 (1%)	15 (1%)	65 (1%)	13 (1%)	67 (1%)
Average total household income before tax (£) ^§,†^					
<18 k	5614 (15%)	1464 (19%)	4150 (15%)	805 (16%)	4809 (16%)
18 k~30 k	7309 (20%)	1566 (2%)	5743 (21%)	997 (2%)	6312 (21%)
31 k~51 k	8281 (23%)	1678 (22%)	6603 (24%)	1241 (24%)	7040 (23%)
52 k~100 k	7365 (20%)	1439 (19%)	5926 (21%)	1007 (2%)	6358 (21%)
>100 k	2459 (6%)	442 (6%)	2017 (7%)	350 (7%)	2109 (7%)
Missing	4529 (12%)	1106 (14%)	3423 (12%)	682 (13%)	3847 (13%)
Townsend deprivation index ^§,†,‡^					
Quantile 1 (<−3.6)	7549 (21%)	1438 (19%)	6111 (22%)	1037 (2%)	6512 (21%)
Quantile 2 (−3.6~−2.1)	8351 (23%)	1710 (22%)	6641 (24%)	1123 (22%)	7228 (24%)
Quantile 3 (−2.1~0.6)	9934 (27%)	2134 (28%)	7800 (28%)	1422 (28%)	8512 (28%)
Quantile 4 (>0.6)	9684 (27%)	2402 (31%)	7282 (26%)	1496 (29%)	8188 (27%)
Missing	39 (1%)	11 (1%)	28 (1%)	4 (1%)	35 (1%)
Smoking statue ^§,†,‡^					
Never	12,368 (34%)	2374 (31%)	9994 (36%)	1730 (34%)	10,638 (35%)
Ever/Current	3426 (9%)	680 (9%)	2746 (10%)	594 (12%)	2832 (9%)
Missing	19,763 (55%)	4641 (60%)	15,122 (54%)	2758 (54%)	17,005 (56%)
Alcohol intake status ^§,†,‡^					
Never	1194 (3%)	429 (6%)	765 (3%)	239 (5%)	955 (3%)
Ever/Current	32,848 (92%)	6599 (86%)	26,249 (94%)	4554 (90%)	28,294 (93%)
Missing	1515 (4%)	667 (9%)	848 (3%)	289 (6%)	1226 (4%)
Ethnic background ^§,†^					
White	32,470 (91%)	6496 (84%)	25,974 (93%)	4677 (92%)	27,793 (91%)
Others	2940 (8%)	1156 (15%)	1784 (6%)	385 (8%)	2555 (8%)
Missing	147 (1%)	43 (1%)	104 (1%)	20 (1%)	127 (1%)
Education achievement ^§,†^					
O levels or equivalent	10,213 (28%)	2522 (33%)	7691 (28%)	1517 (30%)	8696 (29%)
A levels or equivalent	2210 (6%)	464 (6%)	1746 (6%)	331 (7%)	1879 (6%)
University	22,823 (64%)	4608 (6%)	18,215 (65%)	3185 (63%)	19,638 (64%)
Missing	311 (1%)	101 (1%)	210 (1%)	49 (1%)	262 (1%)
Body mass index (BMI; kg/m^2^) ^§,†,‡^					
Normal (<25)	12,041 (33%)	2675 (35%)	9366 (34%)	1541 (30%)	10,500 (34%)
Overweight (25–30)	15,150 (42%)	3128 (41%)	12,022 (43%)	2098 (41%)	13,052 (43%)
Obesity (>30)	8200 (23%)	1846 (24%)	6354 (23%)	1421 (28%)	6779 (22%)
Missing	166 (1%)	46 (1%)	120 (1%)	22 (1%)	144 (1%)
Moderate to vigorous physical activity (MVPA; Metabolic Equivalent Task (MET) minutes/week) ^§,†,‡^					
Quantile 1 (<240)	7609 (21%)	1754 (23%)	5855 (21%)	1222 (24%)	6387 (21%)
Quantile 2 (240–960)	7718 (21%)	1608 (21%)	6110 (22%)	1014 (2%)	6704 (22%)
Quantile 3 (960–2160)	6884 (19%)	1418 (18%)	5466 (2%)	910 (18%)	5974 (2%)
Quantile 4 (>2160)	7379 (20%)	1565 (2%)	5814 (21%)	1042 (21%)	6337 (21%)
Missing	5967 (16%)	1350 (18%)	4617 (17%)	894 (18%)	5073 (17%)
Sleep duration (hour) ^§,†,‡^					
<7 h	9035 (25%)	2027 (26%)	7008 (25%)	1474 (29%)	7561 (25%)
7 h	14,326 (40%)	2916 (38%)	11,410 (41%)	1930 (38%)	12,396 (41%)
8 h	9879 (27%)	2168 (28%)	7711 (28%)	1342 (26%)	8537 (28%)
>8 h	2317 (6%)	584 (8%)	1733 (6%)	336 (7%)	1981 (7%)
Diabetes at baseline ^§,†^					
No	34,104 (95%)	7342 (95%)	26,762 (96%)	4861 (96%)	29,243 (96%)
Yes	1453 (4%)	353 (5%)	1100 (4%)	221 (4%)	1232 (4%)
Cardiovascular diseases at baseline ^§^					
No	33,486 (94%)	7229 (94%)	26,257 (94%)	4788 (94%)	28,698 (94%)
Yes	2071 (5%)	466 (6%)	1605 (6%)	294 (6%)	1777 (6%)
Hypertension at baseline ^§,†^					
No	26,519 (74%)	5668 (74%)	20,851 (75%)	3835 (75%)	22,684 (74%)
Yes	9038 (25%)	2027 (26%)	7011 (25%)	1247 (25%)	7791 (26%)
Healthy diet ^§,†,‡^					
No	7662 (21%)	1774 (23%)	5888 (21%)	1242 (24%)	6420 (21%)
Yes	27,895 (78%)	5921 (77%)	21,974 (79%)	3840 (76%)	24,055 (79%)
Habitual intake of sweeten beverages or foods ^§,†,‡^					
No	886 (2%)	284 (4%)	602 (2%)	161 (3%)	725 (2%)
Yes	34,671 (97%)	7411 (96%)	27,260 (98%)	4921 (97%)	29,750 (98%)
Serum high density liptein (HDL) cholesterol level ^§,†,‡^					
Abnormal	3379 (9%)	764 (10%)	2615 (9%)	562 (11%)	2817 (9%)
Normal	27,872 (78%)	5946 (77%)	21,926 (79%)	3907 (77%)	23,965 (79%)
Missing	4306 (12%)	985 (13%)	3321 (12%)	613 (12%)	3693 (12%)
Serum low density liptein (LDL) cholesterol level ^§,†^					
Abnormal	14,181 (39%)	3276 (43%)	10,905 (39%)	1982 (39%)	12,199 (40%)
Normal	18,507 (52%)	3759 (49%)	14,748 (53%)	2684 (53%)	15,823 (52%)
Missing	2869 (8%)	660 (9%)	2209 (8%)	416 (8%)	2453 (8%)
Spherical equivalent (SE) (Diopters) ^#^					
	−0.06 (1.91)	−0.07 (1.87)	−0.06 (1.93)	−0.08 (1.90)	−0.06 (1.92)
Intraocular pressure (IOP) (mmHg) ^#,†^					
	15.20 (2.93)	15.09 (2.98)	15.23 (2.92)	15.15 (2.94)	15.21 (2.93)

^§^ Categorical variables were presented in the form of “number (percentage)” and tested by the Chi-square test; ^#^ continuous variables were presented in the form of “mean (standard deviation)” and tested by the analysis of variance; ^†^ *p* < 0.05 between coffee drinkers and those who did not drink coffee; ^‡^ *p* < 0.05 between tea drinkers and those who did not drink tea.

**Table 2:** Association between coffee and tea consumption with the average thickness of macular retinal fiber never layer in univariable and multivariable linear regression models.

Multivariable Model 1 ^§^				Multivariable Model 2 ^§^			
Categories (Cups/Day)	Number (No.)	Coefficient (β)	95% Confidential Intervals	Categories (Cups/Day)	Number (No.)	Coefficient (β)	95% Confidential Intervals
Coffee (*p* for trend = 0.88)				Coffee (*p* for trend =0.60)			
0	7695	Reference	Reference	0	7695	Reference	Reference
0.5–1	10,268	0.05	(−0.07~0.18)	0.5–1	10,268	0.03	(−0.09~0.15)
2–3	11,034	0.08	(−0.04~0.12)	2–3	11,034	0.06	(−0.07~0.18)
≥4	6560	−0.02	(−0.17~0.12)	≥4	6560	0.02	(−0.13~0.16)
All	27,862	0.05	(−0.06~0.15)	All	27,862	0.03	(−0.07~0.14)
Tea (*p* for trend = 0.04)				Tea (*p* for trend =0.05)			
0	5082	Reference	Reference	0	5082	Reference	Reference
0.5–1	4309	0.17	(−0.01~0.34)	0.5–1	4309	0.14	(−0.03~0.31)
2–3	10,503	0.12	(−0.03~0.27)	2–3 ^†^	10,503	0.11	(−0.03~0.25)
≥4	15,663	0.16	(0.01~0.30)	≥4 ^†^	15,663	0.15	(0.01~0.29)
All	30,475	0.17	(0.04~0.29)	All ^†^	30,475	0.14	(0.01~0.26)

^§^ Model 1 adjusted for age at baseline, sex, assessment center, average total household income before tax, Townsend deprivation index, ethnic background, education achievement; Model 2 adjusted for smoking statue, drinking statue, body mass index, moderate to vigorous physical activity time, sleep duration, diabetes, cardiovascular diseases, hypertension, healthy diet, habitual intake of sweeten beverages or foods, serum high density lipoprotein cholesterol level, serum low density lipoprotein cholesterol level, spherical equivalent, intraocular pressure, and all covariates in Model 1. Both coffee consumption and tea consumption were included in the multivariable model. ^†^ *p* < 0.05 for coefficients in linear regression models.

**Table 3:** Association between coffee and tea consumption with the average thickness of macular retinal fiber never layer in multivariable linear regression models after further adjusting for the habitual intake of instant coffee.

Multivariable Model 3 ^§^				Multivariable Model 3 ^§^			
Categories (Cups/Day)	Number (No.)	Coefficient (β)	95% Confidential Intervals	Categories (Cups/Day)	Number (No.)	Coefficient (β)	95% Confidential Intervals
Coffee (*p* for trend = 0.07)				Interaction effect (Coffee × Tea) ^‡^			
0	7695	Reference	Reference	C^0^ T^0^	833	Reference	Reference
0.5–1	10,268	0.12	(−0.01~0.26)	C^0^ T^0.5–1^	468	0.43	(−0.04~0.89)
2–3 ^†^	11,034	0.16	(0.03~0.30)	C^0^ T^2–3^	1763	0.32	(−0.02~0.66)
≥4	6560	0.14	(−0.02~0.30)	C^0^ T^≥4^	4631	0.30	(−0.01~0.60)
All ^†^	27,862	0.13	(0.01~0.25)	C^0.5–1^ T^0^	514	0.28	(−0.18~0.73)
				C^0.5–1^ T^0.5–1^	994	0.39	(0.02~0.76)
Tea (*p* for trend = 0.03)				C^0.5–1^ T^2–3^	3068	0.41	(0.10~0.72)
0	5082	Reference	Reference	C^0.5–1^ T^≥4^	5692	0.44	(0.13~0.75)
0.5–1	4309	0.13	(−0.03~0.30)	C^2–3^ T^0^	1437	0.43	(0.07~0.79)
2–3	10,503	0.11	(−0.04~0.25)	C^2–3^ T^0.5–1^	1610	0.40	(0.04~0.75)
≥4 ^†^	15,663	0.15	(0.01~0.29)	C^2–3^ T^2–3^	4172	0.41	(0.09~0.72)
All ^†^	30,475	0.13	(0.01~0.26)	C^2–3^ T^≥4^	3815	0.47	(0.15~0.78)
				C^≥4^ T^0^	2298	0.32	(−0.01~0.64)
Instant coffee				C^≥4^ T^0.5–1^	1237	0.51	(0.15~0.87)
No	21,057	Reference	Reference	C^≥4^ T^2–3^	1500	0.38	(0.02~0.74)
Yes ^†^	14,500	−0.19	(−0.29~−0.10)	C^≥4^ T^≥4^	1525	0.45	(0.09~0.81)

^§^ Model 3 adjusted for age at baseline, sex, assessment center, average total household income before tax, Townsend deprivation index, smoking statue, drinking statue, ethnic background, education achievement, body mass index, moderate to vigorous physical activity time, sleep duration, diabetes, cardiovascular diseases, hypertension, healthy diet, habitual intake of sweeten beverages or foods, serum high density lipoprotein cholesterol level, serum low density lipoprotein cholesterol level, spherical equivalent, intraocular pressure, habitual intake of instant coffee. Both coffee consumption and tea consumption were included in the multivariable model; ^†^ *p* < 0.05 for coefficients in linear regression models; ^‡^ *p* for interaction > 0.05.

## Data Availability

All data used in this study are made available by UK Biobank at http://www.ukbiobank.ac.uk (accessed on 1 December 2022) via data access procedures. Permission to use the UK Biobank Resource was obtained via material transfer agreement as part of Application 62443, 62489, 62491 and 62525.

## Supplementary Materials

The following supporting information can be downloaded at: https://www.mdpi.com/article/10.3390/nu15051196/s1. Table S1: Definition of variables in touchscreen questionnaire, verbal interview, and inpatient records of diagnosis. Table S2: Baseline characteristics in participants with different daily amounts of coffee and tea consumption. Table S3: Association between coffee and tea consumption with the average thickness of macular retinal fiber never layer according to age subgroups. Table S4: Association between coffee and tea consumption with the average thickness of macular retinal fiber never layer in different gender subgroups. Table S5: Baseline characteristics in participants who drank and did not drank instant coffee [7,43].

## References

[1] Landais E., Moskal A., Mullee A., Nicolas G., Gunter M.J., Huybrechts I., Overvad K., Roswall N., Affret A., Fagherazzi G. (2018). Coffee and Tea Consumption and the Contribution of Their Added Ingredients to Total Energy and Nutrient Intakes in 10 European Countries: Benchmark Data from the Late 1990s. Nutrients.

[2] International Coffee Organization World Coffee Consumption. https://www.ico.org/trade_statistics.asp?section=Statistics.

[3] International Tea Committee Annual Bulletin Statistics. https://inttea.com/publications/.

[4] Spaeth A.M., Goel N., Dinges D.F. (2014). Cumulative neurobehavioral and physiological effects of chronic caffeine intake: Individual differences and implications for the use of caffeinated energy products. Nutr. Rev..

[5] Fukutomi R., Ohishi T., Koyama Y., Pervin M., Nakamura Y., Isemura M. (2021). Beneficial Effects of Epigallocatechin-3-O-Gallate, Chlorogenic Acid, Resveratrol, and Curcumin on Neurodegenerative Diseases. Molecules.

[6] Hu G., Bidel S., Jousilahti P., Antikainen R., Tuomilehto J. (2007). Coffee and tea consumption and the risk of Parkinson’s disease. Mov. Disord..

[7] Zhang Y., Yang H., Li S., Li W.D., Wang Y. (2021). Consumption of coffee and tea and risk of developing stroke, dementia, and poststroke dementia: A cohort study in the UK Biobank. PLoS Med..

[8] Yoon J.J., Danesh-Meyer H.V. (2019). Caffeine and the eye. Surv. Ophthalmol..

[9] Haller S., Montandon M.L., Rodriguez C., Herrmann F.R., Giannakopoulos P. (2018). Impact of Coffee, Wine, and Chocolate Consumption on Cognitive Outcome and MRI Parameters in Old Age. Nutrients.

[10] Pham K., Mulugeta A., Zhou A., O’Brien J.T., Llewellyn D.J., Hypponen E. (2021). High coffee consumption, brain volume and risk of dementia and stroke. Nutr. Neurosci..

[11] Zhang S., Otsuka R., Nishita Y., Nakamura A., Kato T., Iwata K., Tange C., Tomida M., Ando F., Shimokata H. (2021). Green tea consumption is associated with annual changes in hippocampal volumes: A longitudinal study in community-dwelling middle-aged and older Japanese individuals. Arch. Gerontol. Geriatr..

[12] Kashani A.H., Asanad S., Chan J.W., Singer M.B., Zhang J., Sharifi M., Khansari M.M., Abdolahi F., Shi Y., Biffi A. (2021). Past, present and future role of retinal imaging in neurodegenerative disease. Prog. Retin. Eye Res..

[13] Jeevakumar V., Sefton R., Chan J., Gopinath B., Liew G., Shah T.M., Siette J. (2022). Association between retinal markers and cognition in older adults: A systematic review. BMJ Open.

[14] Shin J.W., Sung K.R., Song M.K. (2020). Ganglion Cell-Inner Plexiform Layer and Retinal Nerve Fiber Layer Changes in Glaucoma Suspects Enable Prediction of Glaucoma Development. Am. J. Ophthalmol..

[15] Ucak T., Alagoz A., Cakir B., Celik E., Bozkurt E., Alagoz G. (2016). Analysis of the retinal nerve fiber and ganglion cell—Inner plexiform layer by optical coherence tomography in Parkinson’s patients. Park. Relat. Disord..

[16] Santos C.Y., Johnson L.N., Sinoff S.E., Festa E.K., Heindel W.C., Snyder P.J. (2018). Change in retinal structural anatomy during the preclinical stage of Alzheimer’s disease. Alzheimers Dement. (Amst.).

[17] den Haan J., Verbraak F.D., Visser P.J., Bouwman F.H. (2017). Retinal thickness in Alzheimer’s disease: A systematic review and meta-analysis. Alzheimers Dement. (Amst.).

[18] Cheung C.Y., Ikram M.K., Chen C., Wong T.Y. (2017). Imaging retina to study dementia and stroke. Prog. Retin. Eye Res..

[19] Chan M.P., Grossi C.M., Khawaja A.P., Jennifer L.Y., Khaw K.-T., Patel P.J., Khaw P.-T., Morgan J.E., Vernon S.A., Foster P.J. (2016). Associations with Intraocular Pressure in a Large Cohort: Results from the UK Biobank. Ophthalmology.

[20] Yang Q., Reisman C.A., Wang Z., Fukuma Y., Hangai M., Yoshimura N., Tomidokoro A., Araie M., Raza A., Hood N.C. (2010). Automated layer segmentation of macular OCT images using dual-scale gradient information. Opt. Express.

[21] Craig C.L., Marshall A.L., Sjöström M.E., Bauma A.E.L., Booth M.L.E., Ainsworth B.E. (2003). International physical activity questionnaire: 12-country reliability and validity. Med. Sci. Sports Exerc..

[22] Kim H.J., Cho S., Jacobs D.R., Park K. (2014). Instant coffee consumption may be associated with higher risk of metabolic syndrome in Korean adults. Diabetes Res. Clin. Pract..

[23] Qi H., Li S. (2014). Dose-response meta-analysis on coffee, tea and caffeine consumption with risk of Parkinson’s disease. Geriatr. Gerontol. Int..

[24] Matsushita N., Nakanishi Y., Watanabe Y., Kitamura K., Kabasawa K., Takahashi A., Saito T., Kobayashi R., Takachi R., Oshiki R. (2021). Association of coffee, green tea, and caffeine with the risk of dementia in older Japanese people. J. Am. Geriatr. Soc..

[25] Bae J.H., Kim J.M., Lee J.M., Song J.E., Lee M.Y., Chung P.-W., Park K.H. (2020). Effects of consumption of coffee, tea, or soft drinks on open-angle glaucoma: Korea National Health and Nutrition Examination Survey 2010 to 2011. PLoS ONE.

[26] Wu C.M., Wu A.M., Tseng V.L., Yu F., Coleman A.L. (2018). Frequency of a diagnosis of glaucoma in individuals who consume coffee, tea and/or soft drinks. Br. J. Ophthalmol..

[27] Li X., Cheng S., Cheng J., Wang M., Zhong Y., Yu A.Y. (2022). Habitual coffee consumption increases risk of primary open-angle glaucoma: A Mendelian randomization study. Ophthalmology.

[28] Kim J., Aschard H., Kang J.H., Lentjes M.A.H., Do R., Wiggs J.L., Khawaja A.P., Pasquale L.R., Modifiable Risk Factors for Glaucoma Collaboration (2021). Intraocular Pressure, Glaucoma, and Dietary Caffeine Consumption: A Gene-Diet Interaction Study from the UK Biobank. Ophthalmology.

[29] Yilmaz Tugan B., Subasi S., Pirhan D., Karabas L., Yuksel N., Kucuk K.D. (2022). Evaluation of macular and peripapillary vascular parameter change in healthy subjects after caffeine intake using optical coherence tomography angiography. Indian J. Ophthalmol..

[30] Okuno T., Sugiyama T., Tominaga M., Kojima S., Ikeda T. (2002). Effects of caffeine on microcirculation of the human ocular fundus. Jpn. J. Ophthalmol..

[31] Hirata Y. (2021). Trans-Fatty Acids as an Enhancer of Inflammation and Cell Death: Molecular Basis for Their Pathological Actions. Biol. Pharm. Bull..

[32] Liu Z.-M., Tse L.A., Chen B., Wu S., Chan D., Kowk T., Woo J., Xiang Y.-T., Wong S.Y.-S. (2017). Dietary acrylamide exposure was associated with mild cognition decline among non-smoking Chinese elderly men. Sci. Rep..

[33] Garey J., Paule M.G. (2010). Effects of chronic oral acrylamide exposure on incremental repeated acquisition (learning) task performance in Fischer 344 rats. Neurotoxicol. Teratol..

[34] Zhang L., Cao J., Yang H., Pham P., Khan U., Brown B., Wang Y., Zieneldien T., Cao C. (2022). Commercial and Instant Coffees Effectively Lower Abeta1-40 and Abeta1-42 in N2a/APPswe Cells. Front. Nutr..

[35] Mancini R.S., Wang Y., Weaver D.F. (2018). Phenylindanes in Brewed Coffee Inhibit Amyloid-Beta and Tau Aggregation. Front. Neurosci..

[36] Zhang Y., Yang H., Li S., Cao Z., Li W.-D., Yan T., Wang Y. (2021). Association of coffee and genetic risk with incident dementia in middle-aged and elderly adults. Nutr. Neurosci..

[37] Kwon H.S., Koh S.H. (2020). Neuroinflammation in neurodegenerative disorders: The roles of microglia and astrocytes. Transl. Neurodegener..

[38] Madeira M.H., Ortin-Martinez A., Nadal-Nicolas F., Ambrosio A.F., Vidal-Sanz M., Agudo-Barriuso M., Santiago A.R. (2016). Caffeine administration prevents retinal neuroinflammation and loss of retinal ganglion cells in an animal model of glaucoma. Sci. Rep..

[39] Zhang M., Wang L., Wen D., Ren C., Chen S., Zhang Z., Hu L., Yu Z., Tombran-Tink J., Zhang X. (2021). Neuroprotection of retinal cells by Caffeic Acid Phenylethyl Ester (CAPE) is mediated by mitochondrial uncoupling protein UCP2. Neurochem. Int..

[40] Yang Y., Xu C., Chen Y., Liang J.-J., Xu Y., Chen S.-L., Huang S., Yang Q.-C., Cen L.-P., Pang C.P. (2019). Green Tea Extract Ameliorates Ischemia-Induced Retinal Ganglion Cell Degeneration in Rats. Oxid. Med. Cell. Longev..

[41] Yamakuchi M., Bao C., Ferlito M., Lowenstein C.J. (2008). Epigallocatechin gallate inhibits endothelial exocytosis. Biol. Chem..

[42] Jang H.J., Ridgeway S.D., Kim J.A. (2013). Effects of the green tea polyphenol epigallocatechin-3-gallate on high-fat diet-induced insulin resistance and endothelial dysfunction. Am. J. Physiol. Endocrinol. Metab..

[43] Mozaffarian D. (2016). Dietary and Policy Priorities for Cardiovascular Disease, Diabetes, and Obesity: A Comprehensive Review. Circulation.

