# Supplementary material for: Consumption of Coffee and Tea Is Associated with Macular Retinal Nerve Fiber Layer Thickness: Results from the UK Biobank
Source: Nutrients. 2023 Feb 27;15(5):1196. doi: 10.3390/nu15051196 (PMC10005476; doi:10.3390/nu15051196)
Supplement: Supplementary file 1 [file nutrients-15-01196-s001.zip › nutrients-2121370-supplementary.pdf]

Table S1. Definition of variables in touchscreen questionnaire, verbal interview and inpatient records of diagnosis.

Variable	Data category	Data filed	Data code
**Age at recruitment**	Population characteristics	21022	
**Sex**	Population characteristics	31	
**UK Biobank assessment center**	Recruitment	54	
**Average total household income before tax**	Touchscreen questionnaire	738	
**Townsend deprivation index**	Population characteristics	189	
**Smoking status**	Touchscreen questionnaire	20116	
**Alcohol drinker status**	Touchscreen questionnaire	20117	
**Ethnic background**	Touchscreen questionnaire	21000	
**Qualifications**	Touchscreen questionnaire	6138	
**BMI (Body mass index)**	Physical measures	21001	
**MET minutes per week for moderate activity**	Touchscreen questionnaire	22038	
**MET minutes per week for vigorous activity**	Touchscreen questionnaire	22039	
**Sleep duration**	Touchscreen questionnaire	1160	
**Cooked vegetable intake ***	Touchscreen questionnaire	1289	
**Salad / raw vegetable intake ***	Touchscreen questionnaire	1299	
**Fresh fruit intake ***	Touchscreen questionnaire	1309	
**Dried fruit intake ***	Touchscreen questionnaire	1319	
**Oily fish intake ***	Touchscreen questionnaire	1329	
**Non-oily fish intake ***	Touchscreen questionnaire	1339	
**Processed meat intake ***	Touchscreen questionnaire	1349	
**Poultry intake ***	Touchscreen questionnaire	1359	
**Beef intake ***	Touchscreen questionnaire	1369	
**Lamb/mutton intake ***	Touchscreen questionnaire	1379	
**Pork intake ***	Touchscreen questionnaire	1389	
**Never eat eggs, dairy, wheat, sugar**	Touchscreen questionnaire	6144	
**Bread intake ***	Touchscreen questionnaire	1438	
**Bread type ***	Touchscreen questionnaire	1448	
**Cereal intake ***	Touchscreen questionnaire	1458	
**Cereal type ***	Touchscreen questionnaire	1468	
**Tea intake**	Touchscreen questionnaire	1488	
**Coffee intake**	Touchscreen questionnaire	1498	
**Coffee type**	Touchscreen questionnaire	1508	
**HDL cholesterol**	Blood assays	30760	
**LDL direct**	Blood assays	30780	
**logMAR, final (left)**	Eye measures	5208	
**logMAR, final (right)**	Eye measures	5201	
**Cylindrical power (left)**	Eye measures	5086	
**Cylindrical power (right)**	Eye measures	5087	
**Spherical power (left)**	Eye measures	5085	
**Spherical power (right)**	Eye measures	5084	
**Intra-ocular pressure,**	Eye measures	5262	

**corneal-compensated (left)**			
**Intra-ocular pressure, corneal-compensated (right)**	Eye measures	5254	
**Average retinal nerve fibre layer thickness (left)**	Eye measures	28500	
**Average retinal nerve fibre layer thickness (right)**	Eye measures	28501	
**Diabetes**	Hospital inpatients (ICD-10)	41270	E11-E14
	Touchscreen questionnaire	6148	1
	Touchscreen questionnaire	2443	1
	Verbal interview	20002	1276, 1220, 1222, 1223, 1468, 1521, 1607
**Cardiovascular diseases**	Hospital inpatients (ICD-10)	41270	I20-I25, I50, I60-I64
	Touchscreen questionnaire	6150	1, 2, 3
	Verbal interview	20002	1074, 1075, 1076, 1081, 1082, 1086, 1491, 1583
**Hypertension**	Hospital inpatients (ICD-10)	41270	I10, I15
	Touchscreen questionnaire	6150	4
	Verbal interview	20002	1065, 1072
**Glaucoma**	Hospital inpatients (ICD-10)	41270	H40, H42
	Touchscreen questionnaire	6148	2
	Verbal interview	20002	1277
**Retinal diseases**	Hospital inpatients (ICD-10)	41270	H30-H36
	Touchscreen questionnaire	6148	1, 5
	Verbal interview	20002	1275, 1281, 1282, 1528
**Multiple sclerosis**	Hospital inpatients (ICD-10)	41270	G35
	Verbal interview	20002	1261
**Neurodegenerative diseases**	Hospital inpatients (ICD-10)	41270	F00-F03, G20, G21, G30, G31, G122, G231-G233, G238, G239
	Verbal interview	20002	1262, 1263

*** Health diet was determined based on previous literatures.**

Reference:

1. **Mozaffarian D. Dietary and Policy Priorities for Cardiovascular Disease, Diabetes, and Obesity: A Comprehensive Review. Circulation. 2016;133(2):187-225.**
2. **Zhang Y, Yang H, Li S, Li WD, Wang Y. Consumption of coffee and tea and risk of developing stroke, dementia, and poststroke dementia: A cohort study in the UK Biobank. PLoS Med. 2021;18(11): e1003830.**

Table S2. Baseline characteristics in participants with different daily amounts of coffee and tea consumption.

Variable	Total	Coffee consumption (cups/day)				Tea consumption (cups/day)			
		0	0.5-1	2-3	≥4	0	0.5-1	2-3	≥4
**All participants**	35557 (100%)	7695 (100%)	10268 (100%)	11034 (100%)	6560 (100%)	5082 (100%)	4309 (100%)	10503 (100%)	15663 (100%)
**Average thickness of macular retinal nerve fiber layer (μm) #**									
	28.52 (4.24)	28.47 (4.17)	28.55 (4.30)	28.57 (4.26)	28.42 (4.22)	28.44 (4.25)	28.60 (4.25)	28.50 (4.23)	28.53 (4.25)
**Age at baseline § † ‡**									
**<49**	9844 (27%)	2554 (33%)	2673 (26%)	2802 (25%)	1815 (28%)	1721 (34%)	1429 (33%)	2956 (28%)	3738 (24%)
**50-54**	5615 (15%)	1300 (17%)	1564 (15%)	1701 (15%)	1050 (16%)	767 (15%)	659 (15%)	1640 (16%)	2549 (16%)
**55-59**	6212 (17%)	1351 (18%)	1799 (18%)	1936 (18%)	1126 (17%)	837 (16%)	705 (16%)	1792 (17%)	2878 (18%)
**60-64**	8146 (22%)	1451 (19%)	2464 (24%)	2686 (24%)	1545 (24%)	1065 (21%)	897 (21%)	2413 (23%)	3771 (24%)
**>64**	5740 (16%)	1039 (14%)	1768 (17%)	1909 (17%)	1024 (16%)	692 (14%)	619 (14%)	1702 (16%)	2727 (17%)
**Sex § †**									
**Male**	18952 (53%)	4405 (57%)	5774 (56%)	5701 (52%)	3072 (47%)	2761 (54%)	2265 (53%)	5648 (54%)	8278 (53%)
**Female**	16605 (46%)	3290 (43%)	4494 (44%)	5333 (48%)	3488 (53%)	2321 (46%)	2044 (47%)	4855 (46%)	7385 (47%)
**Assessment center § † ‡**									
**Sheffield**	9571 (26%)	1946 (25%)	2616 (25%)	2923 (26%)	2086 (32%)	1448 (28%)	933 (22%)	2495 (24%)	4695 (3%)
**Liverpool**	2628 (7%)	563 (7%)	718 (7%)	770 (7%)	577 (9%)	379 (7%)	251 (6%)	654 (6%)	1344 (9%)
**Hounslow**	6867 (19%)	1506 (2%)	2064 (2%)	2317 (21%)	980 (15%)	918 (18%)	1069 (25%)	2398 (23%)	2482 (16%)
**Croydon**	8935 (25%)	1901 (25%)	2824 (28%)	2853 (26%)	1357 (21%)	1231 (24%)	1267 (29%)	2904 (28%)	3533 (23%)
**Birmingham**	7476 (21%)	1764 (23%)	2023 (2%)	2150 (19%)	1539 (23%)	1093 (22%)	776 (18%)	2030 (19%)	3577 (23%)
**Swansea**	80 (<1%)	15 (<1%)	23 (<1%)	21 (<1%)	21 (<1%)	13 (<1%)	13 (<1%)	22 (<1%)	32 (<1%)
**Average total household income before tax (£) § † ‡**									
**< 18k**	5614 (15%)	1464 (19%)	1636 (16%)	1516 (14%)	998 (15%)	805 (16%)	578 (13%)	1553 (15%)	2678 (17%)
**18k~30k**	7309 (20%)	1566 (2%)	2154 (21%)	2212 (2%)	1377 (21%)	997 (2%)	795 (18%)	2107 (2%)	3410 (22%)
**31k~51k**	8281 (23%)	1678 (22%)	2367 (23%)	2594 (24%)	1642 (25%)	1241 (24%)	984 (23%)	2418 (23%)	3638 (23%)
**52k~100k**	7365 (20%)	1439 (19%)	2073 (2%)	2502 (23%)	1351 (21%)	1007 (2%)	980 (23%)	2313 (22%)	3065 (2%)
**> 100k**	2459 (6%)	442 (6%)	696 (7%)	886 (8%)	435 (7%)	350 (7%)	431 (1%)	820 (8%)	858 (5%)
**Missing**	4529 (12%)	1106 (14%)	1342 (13%)	1324 (12%)	757 (12%)	682 (13%)	541 (13%)	1292 (12%)	2014 (13%)
**Townsend deprivation index § † ‡**									
**Quantile 1 (<-3.6)**	7549 (21%)	1438 (19%)	2128 (21%)	2480 (22%)	1503 (23%)	1037 (2%)	768 (18%)	2172 (21%)	3572 (23%)
**Quantile 2 (-3.6~-2.1)**	8351 (23%)	1710 (22%)	2425 (24%)	2644 (24%)	1572 (24%)	1123 (22%)	874 (2%)	2411 (23%)	3943 (25%)
**Quantile 3 (-2.1~0.6)**	9934 (27%)	2134 (28%)	2883 (28%)	3097 (28%)	1820 (28%)	1422 (28%)	1250 (29%)	2946 (28%)	4316 (28%)
**Quantile 4 (>0.6)**	9684 (27%)	2402 (31%)	2821 (27%)	2801 (25%)	1660 (25%)	1496 (29%)	1411 (33%)	2967 (28%)	3810 (24%)
**Missing**	39 (<1%)	11 (<1%)	11 (<1%)	12 (<1%)	5 (<1%)	4 (<1%)	6 (<1%)	7 (<1%)	22 (<1%)
**Smoking statue § † ‡**									

**Never**	12368 (34%)	2374 (31%)	3574 (35%)	3996 (36%)	2424 (37%)	1730 (34%)	1516 (35%)	3681 (35%)	5441 (35%)
**Ever/Current**	3426 (9%)	680 (9%)	758 (7%)	951 (9%)	1037 (16%)	594 (12%)	463 (11%)	870 (8%)	1499 (1%)
**Missing**	19763 (55%)	4641 (6%)	5936 (58%)	6087 (55%)	3099 (47%)	2758 (54%)	2330 (54%)	5952 (57%)	8723 (56%)
**Drinking statue § † ‡**									
**Never**	1194 (3%)	429 (6%)	271 (3%)	269 (2%)	225 (3%)	239 (5%)	120 (3%)	320 (3%)	515 (3%)
**Ever/Current**	32848 (92%)	6599 (86%)	9607 (94%)	10485 (95%)	6157 (94%)	4554 (9%)	3988 (93%)	9714 (92%)	14592 (93%)
**Missing**	1515 (4%)	667 (9%)	390 (4%)	280 (3%)	178 (3%)	289 (6%)	201 (5%)	469 (4%)	556 (4%)
**Ethnic background § † ‡**									
**White**	32470 (91%)	6496 (84%)	9265 (9%)	10391 (94%)	6318 (96%)	4677 (92%)	3766 (87%)	9194 (88%)	14833 (95%)
**Others**	2940 (8%)	1156 (15%)	961 (9%)	608 (6%)	215 (3%)	385 (8%)	520 (12%)	1269 (12%)	766 (5%)
**Missing**	147 (<1%)	43 (1%)	42 (<1%)	35 (<1%)	27 (<1%)	20 (<1%)	23 (1%)	40 (<1%)	64 (<1%)
**Education achievement § † ‡**									
**O level or equivalent**	10213 (28%)	2522 (33%)	2833 (28%)	2884 (26%)	1974 (3%)	1517 (3%)	999 (23%)	2823 (27%)	4874 (31%)
**A level or equivalent**	2210 (6%)	464 (6%)	695 (7%)	657 (6%)	394 (6%)	331 (7%)	317 (7%)	671 (6%)	891 (6%)
**University**	22823 (64%)	4608 (6%)	6660 (65%)	7414 (67%)	4141 (63%)	3185 (63%)	2951 (68%)	6917 (66%)	9770 (62%)
**Missing**	311 (<1%)	101 (1%)	80 (1%)	79 (1%)	51 (1%)	49 (1%)	42 (1%)	92 (1%)	128 (1%)
**Body mass index (BMI; kg/m^2) § † ‡**									
**Normal (<25)**	12041 (33%)	2675 (35%)	3799 (37%)	3784 (34%)	1783 (27%)	1541 (3%)	1581 (37%)	3706 (35%)	5213 (33%)
**Overweight (25-30)**	15150 (42%)	3128 (41%)	4323 (42%)	4785 (43%)	2914 (44%)	2098 (41%)	1763 (41%)	4453 (42%)	6836 (44%)
**Obesity (>30)**	8200 (23%)	1846 (24%)	2105 (21%)	2414 (22%)	1835 (28%)	1421 (28%)	948 (22%)	2287 (22%)	3544 (23%)
**Missing**	166 (<1%)	46 (1%)	41 (<1%)	51 (<1%)	28 (<1%)	22 (<1%)	17 (<1%)	57 (1%)	70 (<1%)
**Moderate to vigorous physical activity (MVPA; minutes/week) § † ‡**									
**Quantile 1 (<240)**	7609 (21%)	1754 (23%)	2024 (2%)	2269 (21%)	1562 (24%)	1222 (24%)	951 (22%)	2180 (21%)	3256 (21%)
**Quantile 2 (240-960)**	7718 (21%)	1608 (21%)	2301 (22%)	2432 (22%)	1377 (21%)	1014 (2%)	948 (22%)	2456 (23%)	3300 (21%)
**Quantile 3 (960-2160)**	6884 (19%)	1418 (18%)	2028 (2%)	2241 (2%)	1197 (18%)	910 (18%)	883 (2%)	2146 (2%)	2945 (19%)
**Quantile 4 (>2160)**	7379 (20%)	1565 (2%)	2185 (21%)	2313 (21%)	1316 (2%)	1042 (21%)	855 (2%)	2052 (2%)	3430 (22%)
**Missing**	5967 (16%)	1350 (18%)	1730 (17%)	1779 (16%)	1108 (17%)	894 (18%)	672 (16%)	1669 (16%)	2732 (17%)
**Sleep duration (hour) § † ‡**									
**≤6h**	9035 (25%)	2027 (26%)	2509 (24%)	2592 (23%)	1907 (29%)	1474 (29%)	1100 (26%)	2587 (25%)	3874 (25%)
**7h**	14326 (40%)	2916 (38%)	4164 (41%)	4722 (43%)	2524 (38%)	1930 (38%)	1777 (41%)	4333 (41%)	6286 (4%)
**8h**	9879 (27%)	2168 (28%)	2895 (28%)	3095 (28%)	1721 (26%)	1342 (26%)	1177 (27%)	2933 (28%)	4427 (28%)
**≥9h**	2317 (6%)	584 (8%)	700 (7%)	625 (6%)	408 (6%)	336 (7%)	255 (6%)	650 (6%)	1076 (7%)

**Diabetes at baseline § ‡**									
**No**	34104 (95%)	7342 (95%)	9862 (96%)	10614 (96%)	6286 (96%)	4861 (96%)	4108 (95%)	10063 (96%)	15072 (96%)
**Yes**	1453 (4%)	353 (5%)	406 (4%)	420 (4%)	274 (4%)	221 (4%)	201 (5%)	440 (4%)	591 (4%)
**Cardiovascular diseases at baseline § ‡**									
**No**	33486 (94%)	7229 (94%)	9673 (94%)	10429 (95%)	6155 (94%)	4788 (94%)	4090 (95%)	9938 (95%)	14670 (94%)
**Yes**	2071 (5%)	466 (6%)	595 (6%)	605 (5%)	405 (6%)	294 (6%)	219 (5%)	565 (5%)	993 (6%)
**Hypertension at baseline §**									
**No**	26519 (74%)	5668 (74%)	7650 (75%)	8301 (75%)	4900 (75%)	3835 (75%)	3236 (75%)	7794 (74%)	11654 (74%)
**Yes**	9038 (25%)	2027 (26%)	2618 (25%)	2733 (25%)	1660 (25%)	1247 (25%)	1073 (25%)	2709 (26%)	4009 (26%)
**Healthy diet § † ‡**									
**No**	7662 (21%)	1774 (23%)	1972 (19%)	2211 (2%)	1705 (26%)	1242 (24%)	922 (21%)	2011 (19%)	3487 (22%)
**Yes**	27895 (78%)	5921 (77%)	8296 (81%)	8823 (8%)	4855 (74%)	3840 (76%)	3387 (79%)	8492 (81%)	12176 (78%)
**Habitual intake of sweeten beverages or foods § † ‡**									
**No**	886 (2%)	284 (4%)	223 (2%)	257 (2%)	122 (2%)	161 (3%)	106 (2%)	238 (2%)	381 (2%)
**Yes**	34671 (97%)	7411 (96%)	10045 (98%)	10777 (98%)	6438 (98%)	4921 (97%)	4203 (98%)	10265 (98%)	15282 (98%)
**Serum high density liptein (HDL) cholesterol level § † ‡**									
**Abnormal**	3379 (9%)	764 (1%)	876 (9%)	1010 (9%)	729 (11%)	562 (11%)	395 (9%)	975 (9%)	1447 (9%)
**Normal**	27872 (78%)	5946 (77%)	8185 (8%)	8674 (79%)	5067 (77%)	3907 (77%)	3385 (79%)	8259 (79%)	12321 (79%)
**Missing**	4306 (12%)	985 (13%)	1207 (12%)	1350 (12%)	764 (12%)	613 (12%)	529 (12%)	1269 (12%)	1895 (12%)
**Serum low density liptein (LDL) cholesterol level § †**									
**Abnormal**	14181 (39%)	3276 (43%)	4210 (41%)	4253 (39%)	2442 (37%)	1982 (39%)	1730 (4%)	4166 (4%)	6303 (4%)
**Normal**	18507 (52%)	3759 (49%)	5245 (51%)	5892 (53%)	3611 (55%)	2684 (53%)	2221 (52%)	5467 (52%)	8135 (52%)
**Missing**	2869 (8%)	660 (9%)	813 (8%)	889 (8%)	507 (8%)	416 (8%)	358 (8%)	870 (8%)	1225 (8%)
**Spherical equivalent (SE) (Diopters) # † ‡**									
	-0.06 (1.91)	-0.07 (1.87)	-0.06 (1.93)	-0.09 (1.93)	0.01 (1.90)	-0.08 (1.90)	-0.18 (1.94)	-0.08 (1.89)	-0.01 (1.92)
**Intraocular pressure (IOP) (mmHg) # †**									
	15.20 (2.93)	15.09 (2.98)	15.27 (2.91)	15.28 (2.92)	15.09 (2.94)	15.15 (2.95)	15.21 (2.99)	15.24 (2.93)	15.19 (2.92)

§ Categorical variables were presented in the form of “number (percentage)” and tested by the Chi-square test;

# Continuous variables were presented in the form of “mean (standard deviation)” and tested by the analysis of variance;

† P<0.05 among participants with different amounts of coffee consumption;

‡ P<0.05 among participants with different amounts of tea consumption;

Table S3. Association between coffee and tea consumption with the average thickness of macular retinal fiber never layer according to age subgroups

Age at baseline ≤ 60 years old §				Age at baseline > 60 years old §			
**Categories (cups/day)**	**Number (No.)**	**Coefficient (β)**	**95% confidence intervals (95% CI)**	**Categories (cups/day)**	**Number (No.)**	**Coefficient (β)**	**95% confidence intervals (95% CI)**
**Coffee ‡ (P for trend = 0.03)**				**Coffee ‡ (P for trend = 0.88)**			
**0**	5205	Reference	Reference	**0**	2490	Reference	Reference
**0.5-1**	6036	0.14	(-0.03 ~ 0.30)	**0.5-1**	4232	0.08	(-0.14 ~ 0.31)
**2-3 †**	6439	0.20	(0.03 ~ 0.36)	**2-3**	4595	0.09	(-0.14 ~ 0.32)
**≥4**	3991	0.20	(-0.01 ~ 0.40)	**≥4**	2569	0.03	(-0.24 ~ 0.30)
**All**	27,862	0.17	(0.03 ~ 0.32)	**All**	27,862	0.05	(-0.15 ~ 0.26)
**Tea ‡ (P for trend = 0.17)**				**Tea ‡ (P for trend = 0.12)**			
**0**	3325	Reference	Reference	**0**	1757	Reference	Reference
**0.5-1**	2793	0.20	(-0.01 ~ 0.40)	**0.5-1**	1516	0.02	(-0.27 ~ 0.31)
**2-3 †**	6388	0.13	(-0.04 ~ 0.31)	**2-3**	4115	0.07	(-0.18 ~ 0.31)
**≥4 †**	9165	0.14	(-0.03 ~ 0.32)	**≥4**	6498	0.15	(-0.09 ~ 0.39)
**All**	30,475	0.14	(-0.02 ~ 0.29)	**All †**	30,475	0.12	(-0.09 ~ 0.33)

§ Adjusted for age at baseline, sex, assessment center, average total household income before tax, Townsend deprivation index, smoking statue, drinking statue, ethnic background, education achievement, body mass index, moderate to vigorous physical activity time, sleep duration, diabetes, cardiovascular diseases, hypertension, healthy diet, habitual intake of sweeten beverages or foods, serum high density lipoprotein cholesterol level, serum low density lipoprotein cholesterol level, spherical equivalent and intraocular pressure. Both coffee and tea consumption were included in the multivariable model.

‡ P for interaction > 0.05.

Table S4. Association between coffee and tea consumption with the average thickness of macular retinal fiber never layer in different gender subgroups

Female §				Male §			
**Categories (cups/day)**	**Number (No.)**	**Coefficient (β)**	**95% confidence intervals (95% CI)**	**Categories (cups/day)**	**Number (No.)**	**Coefficient (β)**	**95% confidence intervals (95% CI)**
**Coffee ‡ (P for trend = 0.03)**				**Coffee ‡ (P for trend = 0.65)**			
**0**	4405	Reference	Reference	**0**	3290	Reference	Reference
**0.5-1**	5774	0.19	(0.01 ~ 0.37)	**0.5-1**	4494	0.04	(-0.16 ~ 0.24)
**2-3**	5701	0.19	(0.01 ~ 0.37)	**2-3**	5333	0.13	(-0.08 ~ 0.33)
**≥4 †**	3072	0.27	(0.05 ~ 0.49)	**≥4**	3488	0.01	(-0.21 ~ 0.24)
**All**	27,862	0.19	(0.03 ~ 0.35)	**All**	27,862	0.07	(-0.11 ~ 0.25)
**Tea ‡ (P for trend = 0.09)**				**Tea ‡ (P for trend = 0.18)**			
**0**	2761	Reference	Reference	**0**	2321	Reference	Reference
**0.5-1**	2265	0.13	(-0.10 ~0.37)	**0.5-1**	2044	0.16	(-0.08 ~0.41)
**2-3**	5648	0.11	(-0.09 ~ 0.31)	**2-3**	4855	0.13	(-0.08 ~ 0.33)
**≥4**	8278	0.17	(-0.03 ~ 0.36)	**≥4**	7385	0.15	(-0.05 ~ 0.36)
**All**	30,475	0.12	(-0.05 ~ 0.29)	**All †**	30,475	0.16	(-0.02 ~ 0.34)

§ Adjusted for age at baseline, sex, assessment center, average total household income before tax, Townsend deprivation index, smoking statue, drinking statue, ethnic background, education achievement, body mass index, moderate to vigorous physical activity time, sleep duration, diabetes, cardiovascular diseases, hypertension, healthy diet, habitual intake of sweeten beverages or foods, serum high density lipoprotein cholesterol level, serum low density lipoprotein cholesterol level, spherical equivalent and intraocular pressure. Both coffee and tea consumption were included in the multivariable model.

‡ P for interaction > 0.05.

Table S5. Baseline characteristics in participants who drank and did not drank instant coffee.

Variables	Total	Instant coffee drinker	Non-instant coffee drinker
	**35557 (100%)**	**21057(100%)**	**14500 (100%)**
**Average thickness of macular retinal nerve fiber layer (μm) # †**			
	28.52 (4.25)	28.36 (4.25)	28.63 (4.24)
**Age at baseline § †**			
**<49**	9844 (27%)	6119 (29%)	3725 (25%)
**50-54**	5615 (15%)	3351 (15%)	2264 (15%)
**55-59**	6212 (17%)	3726 (17%)	2486 (17%)
**60-64**	8146 (22%)	4683 (22%)	3463 (23%)
**>64**	5740 (16%)	3178 (15%)	2562 (17%)
**Sex § †**			
**Female**	18952 (53%)	11773 (55%)	7179 (49%)
**Male**	16605 (46%)	9284 (44%)	7321 (50%)
**Assessment center § †**			
**Sheffield**	9571 (26%)	5082 (24%)	4489 (30%)
**Liverpool**	2628 (7%)	1382 (6%)	1246 (8%)
**Hounslow**	6867 (19%)	4567 (21%)	2300 (15%)
**Croydon**	8935 (25%)	5890 (27%)	3045 (21%)
**Birmingham**	7476 (21%)	4099 (19%)	3377 (23%)
**Swansea**	80 (<1%)	37 (<1%)	43 (<1%)
**Average total household income before tax (£) § †**			
**< 18k**	5614 (15%)	3087 (14%)	2527 (17%)
**18k~30k**	7309 (20%)	4122 (19%)	3187 (21%)
**31k~51k**	8281 (23%)	4815 (22%)	3466 (23%)
**52k~100k**	7365 (20%)	4587 (21%)	2778 (19%)
**> 100k**	2459 (6%)	1786 (8%)	673 (4%)
**Missing**	4529 (12%)	2660 (12%)	1869 (12%)
**Townsend deprivation index § †**			
**Quantile 1 (<-3.6)**	7549 (21%)	4291 (20%)	3258 (22%)
**Quantile 2 (-3.6~-2.1)**	8351 (23%)	4729 (22%)	3622 (24%)
**Quantile 3 (-2.1~0.6)**	9934 (27%)	5890 (27%)	4044 (27%)
**Quantile 4 (>0.6)**	9684 (27%)	6125 (29%)	3559 (24%)
**Missing**	39 (<1%)	22 (<1%)	17 (<1%)
**Smoking statue § †**			
**Never**	12368 (34%)	7209 (34%)	5159 (35%)
**Ever/Current**	3426 (9%)	1751 (8%)	1675 (11%)
**Missing**	19763 (55%)	12097 (57%)	7666 (52%)
**Alcohol intake status § †**			
**Never**	1194 (3%)	825 (3%)	369 (2%)
**Ever/Current**	32848 (92%)	19133 (90%)	13715 (94%)
**Missing**	1515 (4%)	1099 (5%)	416 (2%)

**Ethnic background § †**			
**White**	32470 (91%)	18997 (90%)	13473 (92%)
**Others**	2940 (8%)	1960 (9%)	980 (6%)
**Missing**	147 (<1%)	100 (<1%)	47 (<1%)
**Education achievement § †**			
**O level or equivalent**	10213 (28%)	5481 (26%)	4732 (32%)
**A level or equivalent**	2210 (6%)	1300 (6%)	910 (6%)
**University**	22823 (64%)	14085 (66%)	8738 (60%)
**Missing**	311 (<1%)	191 (<1%)	120 (<1%)
**Body mass index (BMI; kg/m^2) § †**			
**Normal (<25)**	12041 (33%)	7653 (36%)	4388 (30%)
**Overweight (25-30)**	15150 (42%)	8667 (41%)	6483 (44%)
**Obesity (>30)**	8200 (23%)	4636 (22%)	3564 (24%)
**Missing**	166 (<1%)	101 (<1%)	65 (<1%)
**Moderate to vigorous physical activity (MVPA; minutes/week) § †**			
**Quantile 1 (<240)**	7609 (21%)	4451 (21%)	3158 (21%)
**Quantile 2 (240-960)**	7718 (21%)	4601 (21%)	3117 (21%)
**Quantile 3 (960-2160)**	6884 (19%)	4208 (19%)	2676 (18%)
**Quantile 4 (>2160)**	7379 (20%)	4383 (20%)	2996 (20%)
**Missing**	5967 (16%)	3414 (16%)	2553 (17%)
**Sleep duration (hour) §**			
**≤6h**	9035 (25%)	5305 (25%)	3730 (25%)
**7h**	14326 (40%)	8482 (40%)	5844 (40%)
**8h**	9879 (27%)	5887 (27%)	3992 (27%)
**≥9h**	2317 (6%)	1383 (6%)	934 (6%)
**Diabetes at baseline §**			
**No**	34104 (95%)	20214 (95%)	13890 (95%)
**Yes**	1453 (4%)	843 (4%)	610 (4%)
**Cardiovascular diseases at baseline §**			
**No**	33486 (94%)	19873 (94%)	13613 (93%)
**Yes**	2071 (5%)	1184 (5%)	887 (6%)
**Hypertension at baseline § †**			
**No**	26519 (74%)	15863 (75%)	10656 (73%)
**Yes**	9038 (25%)	5194 (24%)	3844 (26%)
**Healthy diet § †**			
**No**	7662 (21%)	3983 (18%)	3679 (25%)
**Yes**	27895 (78%)	17074 (81%)	10821 (74%)
**Habitual intake of sweeten beverages or foods § †**			
**No**	886 (2%)	594 (2%)	292 (2%)
**Yes**	34671 (97%)	20463 (97%)	14208 (97%)
**Serum high density liptein (HDL) cholesterol level § †**			
**Abnormal**	3379 (9%)	1860 (8%)	1519 (10%)
**Normal**	27872 (78%)	16619 (78%)	11253 (77%)

**Missing**	4306 (12%)	2578 (12%)	1728 (11%)
**Serum low density liptein (LDL) cholesterol level § †**			
**Abnormal**	14181 (39%)	8512 (40%)	5669 (39%)
**Normal**	18507 (52%)	10835 (51%)	7672 (52%)
**Missing**	2869 (8%)	1710 (8%)	1159 (7%)
**Spherical equivalent (SE) (Diopters) # †**			
	-0.06 (1.91)	0.01 (1.90)	-0.11 (1.92)
**Intraocular pressure (IOP) (mmHg) #**			
	15.20 (2.93)	15.21 (2.93)	15.19 (2.93)

§ Categorical variables were presented in the form of “number (percentage)” and tested by the Chi-square test;

# Continuous variables were presented in the form of “mean (standard deviation)” and tested by the analysis of variance;

† P<0.05 between instant coffee drinkers and those who did not drink instant coffee;
